# Involvement of Cancer Stem Cells in Chemoresistant Relapse of Epithelial Ovarian Cancer Identified by Transcriptome Analysis

**DOI:** 10.1155/2022/6406122

**Published:** 2022-03-31

**Authors:** Yaoqi Sun, Lin Yao, Chunyan Wang, Bing Xiong, Jing Guo, Lian Wang, Jihui Zhu, Zhongping Cheng, Shupeng Liu

**Affiliations:** ^1^Department of Obstetrics and Gynecology, Shanghai Tenth People's Hospital, School of Medicine, Tongji University, Shanghai 200072, China; ^2^Nanjing Medical University, Nanjing 211100, China; ^3^Dana-Farber Cancer Institute, Boston, MA 02215, USA; ^4^Department of Obstetrics and Gynecology, Putuo People's Hospital, Tongji University, Shanghai 200060, China

## Abstract

Epithelial ovarian cancer (EOC) is the most lethal gynecological malignancy. Despite the initial resection and chemotherapeutic treatment, relapse is common, which leads to poor survival rates in patients. A primary cause of recurrence is the persistence of ovarian cancer stem cells (OCSCs) with high tumorigenicity and chemoresistance. To achieve a better therapeutic response in EOC relapse, the mechanisms underlying acquired chemoresistance associated with relapse-initiating OCSCs need to be studied. Transcriptomes of both chemosensitive primary and chemoresistant relapse EOC samples were obtained from ICGC OV-AU dataset for differential expression analysis. The upregulated genes were further studied using KEGG and GO analysis. Significantly increased expression of eighteen CSC-related genes was found in chemoresistant relapse EOC groups. Upregulation of the expression in four hub genes including WNT3A, SMAD3, KLF4, and PAX6 was verified in chemoresistant relapse samples via immunohistochemistry staining, which confirmed the existence and enrichment of OCSCs in chemoresistant relapse EOC. KEGG and GO enrichment analysis in microarray expression datasets of isolated OCSCs indicated that quiescent state, increased ability of drug efflux, and enhanced response to DNA damage may have caused the chemoresistance in relapse EOC patients. These findings demonstrated a correlation between OCSCs and acquired chemoresistance and illustrated potential underlying mechanisms of OCSC-initiated relapse in EOC patients. Meanwhile, the differentially expressed genes in OCSCs may serve as novel preventive or therapeutic targets against EOC recurrence in the future.

## 1. Introduction

Epithelial ovarian cancer (EOC) is the most lethal gynecological cancer with a 5-year relative survival rate of 29% in patients at advanced stage [1]. The poor prognosis of EOC is largely attributed to relapse and chemoresistance [2, 3]. More than 70% EOC patients suffered from tumor recurrence after standard treatments including optimal surgery and platinum/paclitaxel chemotherapy [4]. The majority of these patients had to receive frequent chemotherapy or radiotherapy with gradually shortened platinum-free intervals (PFI), which finally led to chemoresistance [5]. Poly ADP-ribose polymerase (PARP) inhibitors such as olaparib have been reported to increase the progress-free survival (PFS) in EOC, prolong PFI, and delay relapse [6]. However, only patients with Breast Cancer susceptibility gene 1/2 (BRCA1/2) mutations or homologous recombination deficiency (HRD), which account for around 34% and 50% of the total patients, respectively, are recommended to use these agents [3, 7, 8]. No therapeutic method was proved effective in treating recurrent chemoresistant EOCs. The underlying mechanisms of relapse and chemoresistance remain unclear.

Cancer stem cells (CSCs) are a subpopulation of cancer cells with the capabilities of self-renewal and differentiation. Emerging evidence has indicated CSCs' role as the seed of tumor development, relapse, and drug resistance in various types of malignancies including EOC [9, 10]. CSCs in EOC were firstly purified from a patient's ascites, which displayed their tumorigenic property [11]. Other ovarian cancer stem cells (OCSCs), identified by surface markers CD44, Prominin 1 (PROM1, CD133), and Aldehyde Dehydrogenase 1 Family Member A1 (ALDH1A1), reportedly involved in EOC development, relapse, and chemoresistance [12]. The inherent characteristics of high plasticity, slow cell cycling, and efficient DNA repair empowered CSCs to survive treatment and enabled further chemoresistance [13]. Increased expression of CSC markers and transcription factors, such as CD44, KIT Proto-Oncogene (KIT, CD117), POU Class 5 Homebox 1 (POU5F1, OCT4), and NANOG, was observed in ovarian cancer cells treated by cisplatin or paclitaxel both *in vitro* and *in vivo* [14–16]. Higher expression level of CSC markers including ALDH1A1, CD44, and CD133 was also reported in tumor tissues collected immediately after primary chemotherapy compared with paired primary samples from EOC patients [17]. In tumors from recurrent platinum-resistant EOC patients, CD133 expression was significantly increased [17]. These findings suggested that OCSCs survived after chemotherapy, generated chemoresistance, and contributed to EOC recurrence. OCSCs are thus considered as a promising therapeutic target against chemoresistant relapse of ovarian cancer. Landen et. al reported that targeting ALDH1A1 in EOC sensitized resistant tumor cells to chemotherapy [18]. Knockdown of GLI Family Zinc Finger 2 (Gli2), a transcription factor in Hedgehog signaling pathway, increased sensitivity of EOC cells to cisplatin [17]. Notably, OCSCs were identified recently using specific surface markers, and a few subpopulations had just been reported in EOC. The mechanisms underlying acquired chemoresistance associated with relapse-initiating OCSCs are not fully understood.

To investigate the molecules and pathways involved in OCSC-induced EOC chemoresistant recurrence, we identified differentially expressed genes (DEGs) between paired chemosensitive primary and chemoresistant relapse samples from EOC patients based on transcriptomic data from next-generation RNA-sequencing analysis. Functional enrichment analysis using DEGs found that signaling pathways regulating pluripotency of stem cells were upregulated in chemoresistant relapse samples. Upregulation of hub gene expression, including Wnt Family Member 3A (WNT3A), SMAD Family Member 3 (SMAD3), Kruppel-Like Factor 4 (KLF4), and Paired Box 6 (PAX6), was validated using immunohistochemistry (IHC) staining in chemoresistant relapse samples. Moreover, we demonstrated that the biological processes (BPs) such as DNA damage response, drug efflux, and quiescent state were enriched in OCSCs compared with non-OCSCs, which may explain the underlying mechanisms of chemoresistance in OCSCs. Our study suggested the involvement of CSCs in EOC chemoresistant relapse using transcriptomic data from clinical tumor samples. Through transcriptome analysis, we found out relevant genes and molecular pathways potentially contributing to chemoresistance in OCSCs. These findings reinforced the role of OCSCs as a promising therapeutic target in management of chemoresistant recurrent EOC and further suggested that these DEGs might become novel preventive or therapeutic targets against OCSCs in the future.

## 2. Materials and Methods

### 2.1. Data Resources

The RNA sequencing data of International Cancer Genome Consortium Ovarian Cancer-Australia (ICGC OV-AU) was obtained from the ICGC Data Portal (https://dcc.icgc.org/) and European Genome-phenome Archive (EGA) repository (EGAD00001000877, https://ega-archive.org/) [19]. Patients who relapse 6 months or more after initial chemotherapy are considered as chemosensitive primary cases, while patients relapse within 6 months are termed as chemoresistant primary cases. Chemoresistant relapse is only for patients who were sensitive to initial chemotherapy but failed subsequent treatment [19, 20]. According to the criterion of chemosensitive primary and chemoresistant relapse, 24 of 93 patients from ICGC OV-AU dataset were included in this study. A total of 39 samples from these 24 EOC patients, including 14 chemosensitive primary samples, 12 paired chemoresistant relapse samples, and 13 unpaired chemoresistant relapse samples, were selected for further analysis. Detailed information of the samples is listed in Supplementary Table S1. A total of 8 microarray datasets, including 5 chemotherapy-related datasets and 3 CSC-related datasets (Supplementary Table S2), were downloaded from the Gene Expression Omnibus (GEO) database (https://www.ncbi.nlm.nih.gov/geo/) [21–27].

### 2.2. Principle Component Analysis (PCA)

To detect the outlying samples, the PCA of the normalized expression matrix was performed, relied on the FactoMineR package (version 2.3) [28]. The normalized expression matrix from the ICGC OV-AU dataset was established by filtering genes with low read abundance after the rlog transformation based on the R package DESeq2 (version 1.28.1) [29]. Finally, the FactoExtra package (version 1.0.7) was used to visualize the results of PCA [30].

### 2.3. Differential Expression Analysis

After the PCA, a total of 36 samples from the ICGC OV-AU were sent for further analysis. The DESeq2 package was applied to identify DEGs between chemosensitive primary and chemoresistant relapse samples. Genes with ∣log_2_ fold change | >1 and adjusted probability (*P*) value < 0.05 were considered statistically significant. The volcano plot was created by ggplot2 (version 3.3.2) [31], and the heatmap (hierarchical clustering, using Euclidean distances and the complete algorithm, scaled by gene) based on the rlog-transformed expression value of these DEGs was generated by the pheatmap package (version 1.0.12) [32]. For the microarray data from GEO database, the limma package (version 3.44.4) was used to analyze changes in gene expression [33].

### 2.4. Kyoto Encyclopedia of Genes and Genomes (KEGG) Pathway and Gene Ontology (GO) Enrichment Analysis

To explore the potential function of DEGs in chemoresistant relapse samples, the clusterProfiler package (version 3.16.1) was used to perform KEGG pathway and GO enrichment analysis, with adjusted *P* value < 0.05 as the threshold [34].

### 2.5. Protein-Protein Interaction (PPI) Network Analysis

The PPI network analysis (medium confidence: minimum required interaction score = 0.400) was performed using STRING database (https://string-db.org/, version 11.0) and exported using Cytoscape 3.7.1.

### 2.6. Ovarian Cancer Samples

10 primary ovarian cancer tissues and 2 recurrent ovarian cancer tissues were obtained from 11 patients who underwent operation in the Shanghai Tenth People's Hospital, School of Medicine, Tongji University, Shanghai, China (Approval No. 2020-KN123-01). The clinical information of these patients is shown in Supplementary Table S3. All subjects signed the informed consents before inclusion.

### 2.7. Immunohistochemistry

Tumor tissues from ovarian cancer patients were fixed by formalin and embedded with paraffin. Sections from paraffin-embedded specimens were dewaxed, dehydrated, and subjected to antigen retrieval. After blocking endogenous peroxidases, the sections were incubated with primary antibody overnight at 4°C. The antibodies were as follows: KLF4 (#ab215036, rabbit monoclonal, Abcam, 1 : 2000), PAX6 (#ab195045, rabbit monoclonal, Abcam, 1 : 500), SMAD3 (#ab40854, rabbit monoclonal, Abcam, 1 : 500), and WNT3A (#ab219412, rabbit monoclonal, Abcam, 1 : 500). Detailed information of the antibodies is listed in Supplementary Table S4. Wash with 1x phosphate-buffered saline (1xPBS), followed by incubation in horseradish peroxidase- (HRP-) conjugated secondary antibody at 37°C for 1 hour and detection using diaminobenzidine (DAB). At least 10 random images from stained sections were captured by a light microscope. The intensity of DAB staining and the percentage of DAB positive cells were analyzed using IHC Profiler in ImageJ [35]. Quantification of KLF4, PAX6, SAMD3, and WNT3A for each sample was determined by H-score ([1 × (%of weak staining) + 2 × (%of moderate staining) + 3 × (%of strong staining)]) [36].

### 2.8. Statistical Analysis

Statistical analysis was performed using GraphPad Prism 8. The parametric Student *t*-test was used for the analysis of H-score between chemosensitive primary and chemoresistant primary samples. The nonparametric Mann-Whitney test was used for the analysis of H-score between chemosensitive primary and chemoresistant relapse samples. Data were shown as mean ± SD. *P* value < 0.05 was considered to be statistically significant.

## 3. Results

### 3.1. Identification of CSC Pathways Enriched in Chemoresistant Relapse EOC Samples

**Figure 1:**
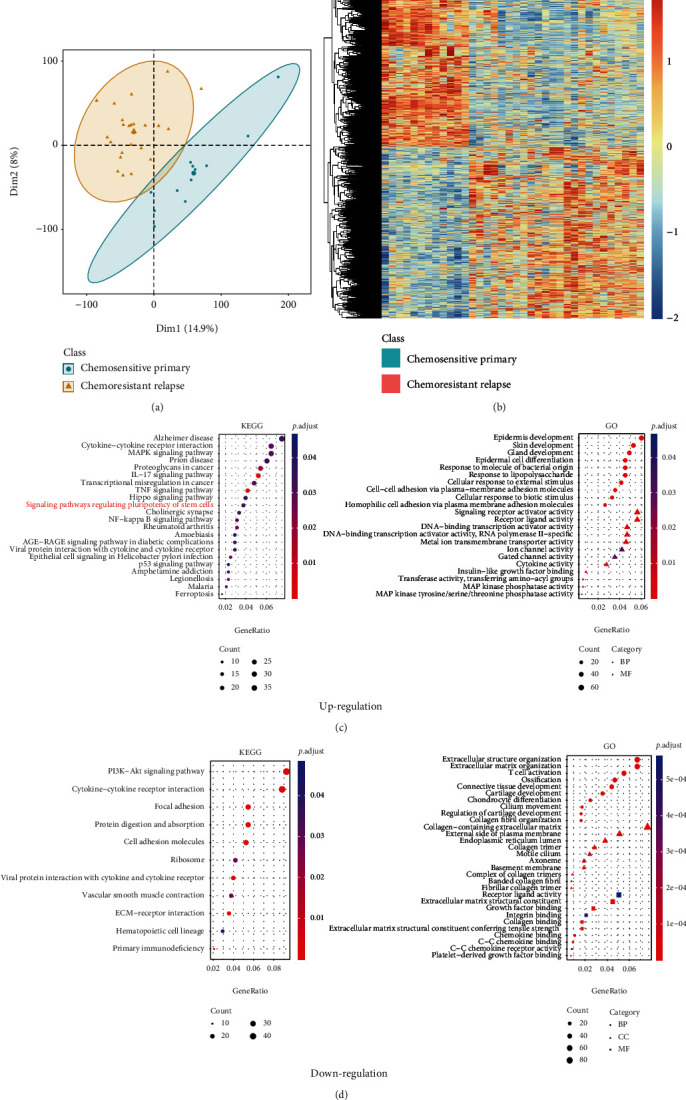
Identification and functional enrichment analysis of DEGs. (a) PCA score plots show 12 chemosensitive primary and 24 chemoresistant relapse samples selected for further analysis. (b) Heatmap of a total of 2835 differentially expressed genes. Red means upregulation while blue means downregulation. (c) Enriched KEGG and GO terms in chemoresistant relapse samples based on DEGs (adjusted *P* value < 0.05). (d) Enriched KEGG and GO terms in chemosensitive primary samples based on DEGs (adjusted *P* value < 0.05).

To investigate the CSC pathways involved in chemoresistance and recurrence of EOC unbiasedly, published transcriptomic data of both chemosensitive primary and chemoresistant relapse EOC samples was obtained from ICGC OV-AU dataset. The samples were clustered into two different classes according to their clinical characteristics of chemosensitivity (Supplementary Figure S1). The global gene expression pattern of all 39 samples was firstly analyzed by PCA. Three out of 39 samples (AOCS-093-10-1, AOCS-094-4-2, and AOCS-137-4-0) showed different gene expression patterns compared to the other samples with similar clinical characteristics. They were thus excluded for further analysis to avoid bias. Further PCA showed that the remaining samples (*n* = 36) were clustered into classes of chemosensitive (*n* = 12) or chemoresistant (*n* = 24) (Figure 1(a)). Then, differential expression analysis was performed to identify the specific gene expression profiles of tumor tissues from different classes. A total of 2835 DEGs including 1389 upregulated and 1446 downregulated genes were identified in chemoresistant relapse tumor samples vs. chemosensitive primary samples (Supplementary Figure S2). The ability to identify chemoresistant relapse samples from chemosensitive primary samples via unsupervised clustering analysis of DEGs (Figure 1(b)) indicated that tumor samples from chemoresistant and chemosensitive groups have different gene expression profiles. KEGG and GO analyses were then performed to investigate the signaling pathways and BPs activated in chemoresistant relapse tumors. KEGG analysis showed that upregulated DEGs in chemoresistant relapse tumors were enriched in 22 KEGG pathways including NF-*κ*B signaling pathway, MAPK signaling pathway, and Hippo signaling pathway (Figure 1(c) and Table 1). Specifically, signaling pathways regulating pluripotency of stem cells were enriched in chemoresistant relapse tumors (adjusted *P* value = 0.0019) (Figure 1(c) and Table 1). GO analysis found that these upregulated DEGs were mainly enriched in BPs involving organ development, such as epidermis development, skin development, gland development, and epidermal cell differentiation (Figure 1(c) and Table 2). And downregulated DEGs in chemoresistant relapse tumors were enriched in PI3K-Akt signaling pathway, ECM-receptor interaction, primary immunodeficiency, and BPs including extracellular matrix organization, and T cell activation (Figure 1(d) and Tables 1 and 2). These data demonstrated that chemoresistant relapse tumors possessed different gene expression pattern compared with chemosensitive primary samples. Further functional enrichment analysis suggested potential activation of signaling pathways regulating pluripotency of stem cells in chemoresistant relapse tumor samples, indicating the involvement of OCSCs in chemoresistant recurrence of EOC.

### 3.2. Identification of Crucial OCSC Genes Involved in Chemoresistant Relapse

**Figure 2:**
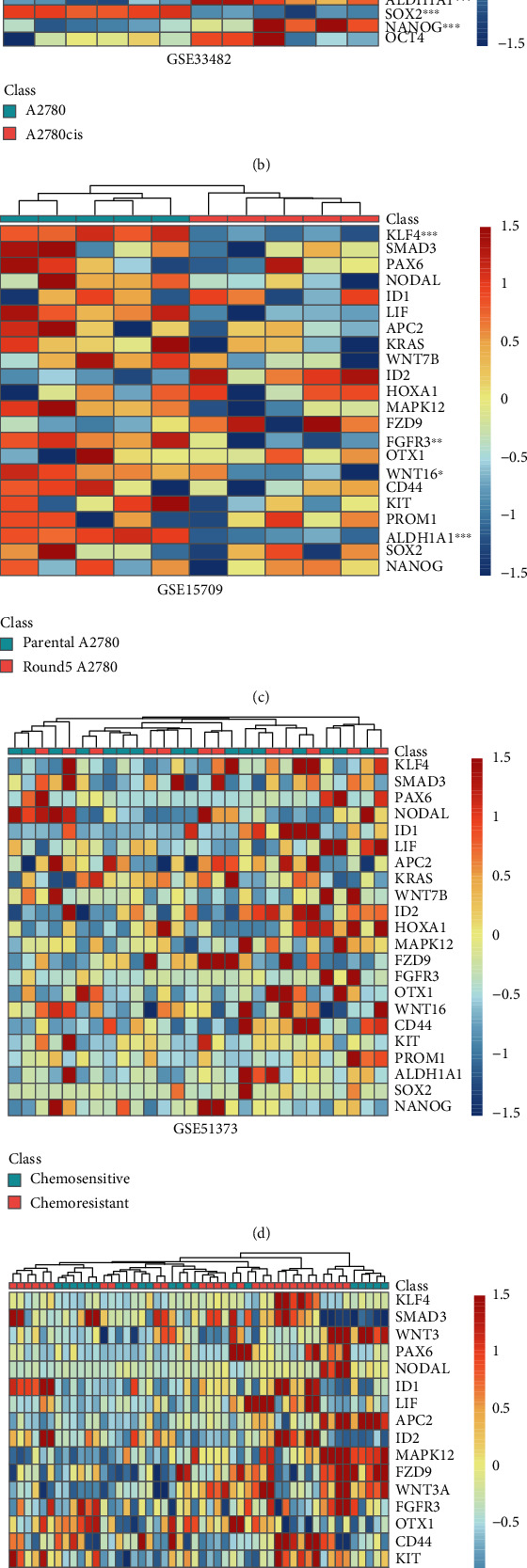
The expression of 18 CSC-related genes and 7 CSC markers in ICGC OV-AU and 5 GEO datasets. (a) ICGC OV-AU, (b) GSE33482, (c) GSE15709, (d) GSE51373, (e) GSE28739, and (f) GSE131978. (^∗^adj.*P* < 0.05, ^∗∗^adj.*P* < 0.01, and ^∗∗∗^adj.*P* < 0.001).

Having found that signaling pathways upregulated in chemoresistant relapse samples, we intended to investigate the genes involved in chemoresistant relapse of EOC. Firstly, DEGs listed in signaling pathways regulating pluripotency of stem cells were investigated. It showed that 18 DEGs in this pathway were upregulated in chemoresistant relapse class (Figure 2(a)). Among them, the expression of KLF4, a well-known stem cell marker [37], was most notably upregulated (log_2_ fold change = 2.93, adjusted *P* value = 2.52e^−10^). Genes in Wnt signaling pathway (WNT3, WNT7B, WNT3A, WNT16, Frizzled Class Receptor 9 (FZD9), and Adenomatous Polyposis Coli 2 (APC2)), TGF-*β* signaling pathway (Nodal Growth Differentiation Factor (NODAL), SMAD3, Inhibitor of DNA Binding 1 (ID1), and ID2), and MAPK signaling pathway (Fibroblast Growth Factor Receptor 3 (FGFR3), KRAS, and Mitogen-Activated Protein Kinase 12 (MAPK12)) were all upregulated to different extents (Supplementary Table S5). In addition, the expression levels of 4 CSC surface markers including CD44, CD117, CD133, and ALDH1A and 3 stem cell transcription factors including SRY-Box Transcription Factor 2 (SOX2), OCT4, and NANOG were also investigated. However, no significant difference was found in the expression of these 7 CSC-related genes between chemoresistant relapse tumor samples and chemosensitive primary samples (Figure 2(a)).

Then, the expression levels of 18 DEGs and 7 CSC-related genes in chemoresistant ovarian cancer cell lines and chemoresistant tumor samples were assessed for further validation. The published microarray expression datasets (GSE33482 and GSE15709 for chemoresistant tumor cells line and GSE51373, GSE28739, and GSE131978 for chemoresistant tumors) were obtained from GEO database. In dataset GSE33482 [38], the expression of WNT3, WNT3A, FGFR3, OTX1, CD117, ALDH1A1, SOX2, and NANOG was elevated in chemoresistant A2780cis line but not its parental chemosensitive line A2780, while the expression of other genes remained unchanged (Figure 2(b)). In dataset GSE15709 [21], the expression of WNT16, KLF4, FGFR3, and ALDH1A1 was remarkably downregulated in chemoresistant Round5 A2780 line compared with the parental line A2780, while other genes showed no statistical difference at the transcriptional level (Figure 2(c)). In datasets for chemoresistant primary tumors (GSE51373 [22], GSE28739 [23], and GSE131978 [24]), none of the 25 CSC-related genes showed notable change between primary chemoresistant samples and primary chemosensitive samples (Figures 2(d)–2(f)).

**Figure 3:**
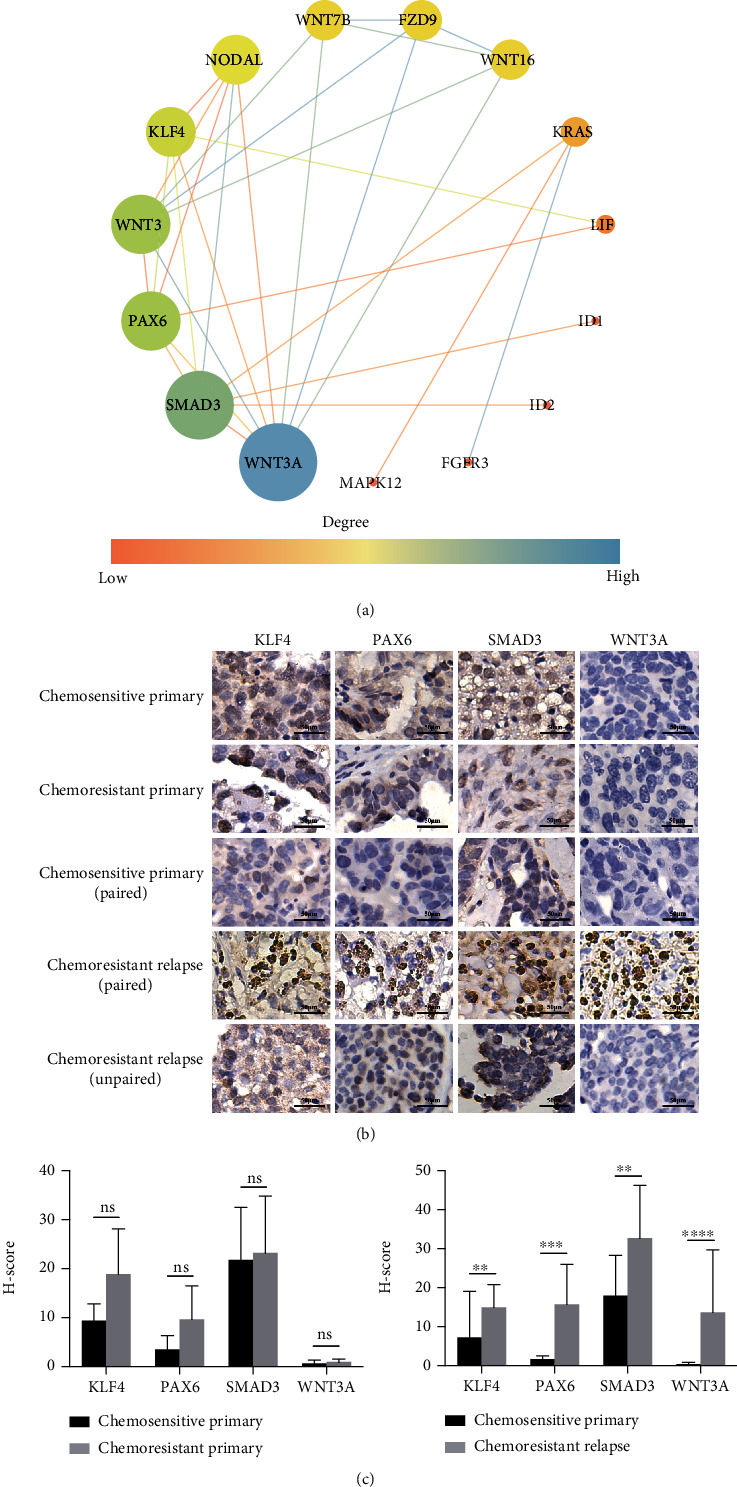
The validation of the CSC-related genes. (a) The PPI network of 18 CSC-related DEGs; the disconnected nodes were hidden. The size of the node represents the degree of the gene, while the size of the edge indicates the combined score of the two interacting genes. Genes without connecting nodes were not shown in the picture. (b) IHC images of tumor tissue samples from chemosensitive primary, chemoresistant primary, and chemoresistant relapse patients. The sections were stained with antibodies specific for KLF4, PAX6, SMAD3, and WNT3A as described in Materials and Methods. Magnification 200x and scale bar = 50 *μ*m. The entire images are shown in Supplementary Figure S3. (c) Expression of KLF4, PAX6, SMAD3, and WNT3A protein quantified by H-score. Bar charts represent mean ± SD (left: chemosensitive primary: *n* = 5 samples, chemoresistant primary: *n* = 4 samples; right: *n* = 10 images; ^∗^*P* < 0.05, ^∗∗^*P* < 0.01, and ^∗∗∗^*P* < 0.001).

Additionally, the expression of CSC-related genes was further assessed in EOC specimens via IHC staining. A total of 12 paraffin-embedded tumor tissue samples (1 pair of chemosensitive primary and chemoresistant relapse, 1 chemoresistant relapse, 5 chemosensitive primary, and 4 chemoresistant primary) were obtained from EOC patients (Supplementary Table S3). The PPI network analysis identified 4 hub genes out of 18 DEGs, including WNT3A/WNT3, SMAD3, PAX6, and KLF4, which were selected for further validation (Figure 3(a)). Positive staining of KLF4 and SMAD3 was observed in paired chemosensitive primary tumor, while the expression of all 4 hub genes was observed in paired chemoresistant relapse tumor with a significant enhanced signal in KLF4 and SMAD3 staining (Figure 3(b)). Signal of KLF4, SMAD3, and PAX6, but not WNT3A, was detected in the unpaired chemoresistant relapse tumors (Figure 3(b)). A similar expression pattern was found in chemosensitive primary and chemoresistant primary tumors with positive staining of KLF4, SMAD3, and PAX6, but negative staining of WNT3A (Figure 3(b)). Statistical analysis demonstrated that the H-score of 4 hub genes was higher in chemoresistant relapse tumor than in the paired chemosensitive primary tumor, which was in consistence with the results of transcriptome analysis (Figure 3(c)). No significant difference was detected in H-score of these 4 hub genes between chemosensitive primary and chemoresistant primary samples (Figure 3(c)). These results revealed that CSC-related genes such as KLF4, PAX6, SMAD3, and WNT3A were correlated with acquired chemoresistance of EOC, while no evidence of direct correlation was found between CSC-related genes and chemoresistance in primary tumors during this study. It suggested that these aberrantly expressed chemoresistant relapse-related CSC genes could be used as potential therapeutic targets in patients suffering from EOC recurrence.

### 3.3. Identification of the Mechanisms Underlying Chemoresistance of OCSCs

**Figure 4:**
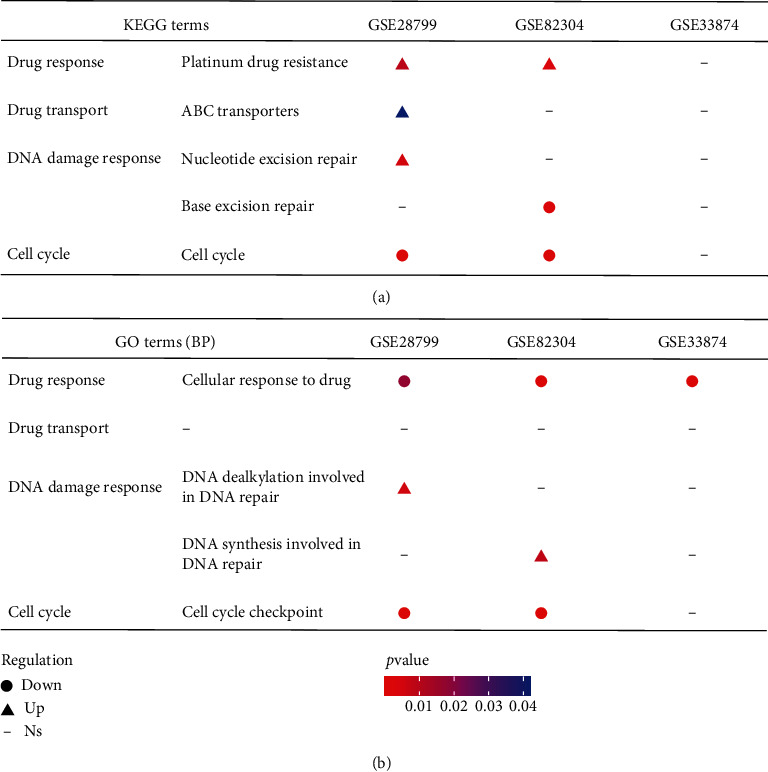
The characteristics of OCSCs related to chemoresistance. (a) The KEGG terms enriched in GSE28799, GSE82304, and GSE33874, with *P* value < 0.05 as the threshold. (b) The GO BP terms enriched in GSE28799, GSE82304, and GSE33874, with *P* value < 0.05 as the threshold.

Recent studies reported that the chemoresistance of OCSCs may be traceable to its quiescent state, increased ability of drug efflux, and enhanced response to DNA damage, all of which might help OCSCs to survive the conventional chemotherapy [10]. To clarify the correlation between these OCSC properties and acquired chemoresistance, 3 microarray expression datasets of isolated OCSCs (GSE28799, GSE82304, and GSE33874) were obtained from GEO database. KEGG and GO enrichment analyses were performed based on DEG information from each dataset, respectively. KEGG pathways and GO BPs correlated with cell cycle, drug response, drug transport, and DNA damage response were listed specifically. KEGG analysis showed that cell cycle pathway was inhibited, while pathways of platinum drug resistance, ABC transporters, and nucleotide excision repair were activated in spheroid OVCAR3 (GSE28799) [26] (Figure 4(a)). Pathways of cell cycle and base excision repair were inhibited in ALDH-high SKOV3 (GSE82304) [25], while platinum drug resistance pathways were activated (Figure 4(a)). In side population (SP) cells from fresh ascites of HGSOC patients (GSE33874) [27], none of the KEGG pathways mentioned above was significantly enriched (Figure 4(a)). GO analysis showed that BPs of cell cycle and drug response were inhibited and DNA damage response was activated in spheroid OVCAR3 (GSE28799) and ALDH-high SKOV3 (GSE82304), while no drug transport in BPs was enriched (Figure 4(b)). In SP cells (GSE33874), BP of drug response was inhibited, while BPs of cell cycle, drug transport, and DNA damage response were not enriched (Figure 4(b)). These results confirmed that quiescent state, increased ability of drug efflux, and enhanced response to DNA damage may have caused the chemoresistant recurrence in EOC patients.

## 4. Discussion

Chemoresistant recurrence accounts for most fatalities in EOC patients. The majority of patients, who were successfully treated with chemotherapy following their diagnosis, suffered from recurrence with shortening PFI, which led to chemoresistance and death eventually [10]. OCSCs were considered the primary cause of tumor relapse and chemoresistance. In present study, we revealed the enrichment of CSCs in chemoresistant relapse compared with chemosensitive primary tumors by analyzing transcriptomic data from EOC patients. CSC-related genes such as KLF4, SMAD3, and PAX6 from signaling pathways regulating pluripotency of stem cells were found upregulated in chemoresistant relapse tumors via bioinformatics analysis and IHC. No significant difference was noticed in the expression of CSC markers including CD44, CD133, and ALDH1A1 or stem cell transcriptional factors such as SOX2, OCT4, and NANOG between chemoresistant relapse and chemosensitive primary samples. By analyzing the microarray expression data of OCSCs via KEGG and GO, we further validated that quiescent state, increased ability of drug efflux, and enhanced response to DNA damage may have caused the chemoresistant recurrence and led to low survival rates in EOC patients.

Enrichment of CSCs had been reported in post chemotherapy tumors via CSC marker identification. Based on the expression of several CSC markers, such as CD44, CD24, and Epithelial cell Adhesion Molecule (EpCAM), a population of cells, characterized with increased tumorigenic, metastatic, and chemoresistant potential, was found enriched *in vitro* culture after the treatment of cisplatin or paclitaxel [16]. CD133^+^ and Stem cell antigen-1^+^ (Sca-1^+^) tumor-initiating cells were also detected to persist after paclitaxel and carboplatin chemotherapy in mouse models [39]. In our study, we revealed 18 pivotal CSC-related genes upregulated in chemoresistant relapse samples through transcriptomics. Several frequently used CSC identification markers including CD44, CD117, CD133, ALDH1A, SOX2, OCT4, and NANOG were also investigated between chemoresistant relapse and chemosensitive primary samples. These genes were reported to be overexpressed in and be associated with the progression of many malignant tumors such as melanoma, bladder cancer, prostate cancer, gastric cancer, and colorectal cancer [40–45]. However, the expression levels of these genes were found with no statistical difference, which may be ascribed to the phenotypic and functional heterogeneity of CSCs. For example, ALDH^+^ CSCs tend to be more proliferative, the CD44^+^/CD24^−^ CSCs have a disposition to be more invasive, and the Leucine Rich Repeat Containing G Protein-Coupled Receptor 5^+^ (LGR5^+^) cells are more quiescent and chemoresistant [46, 47]. Therefore, the heterogeneity of CSC markers is one of the reasons for the inefficiency of CSC targeting. In addition, it was reported that posttranscriptional mechanisms and proteostasis might cause inconsistent expression of CSC markers between mRNA and protein levels [48]. Although we found no significant difference of these CSC markers according to the transcriptome analysis, there might be remarkable differences at the protein level due to these epigenetic regulations.

The 18 DEGs detected in this study are mainly involved in Wnt, TGF-*β*, and MAPK pathways, which may become potential therapeutic targets to eliminate OCSCs (Figure 2). Moreover, 4 out of these 18 identified genes were further confirmed in resected tissues from EOC patients through transcriptome analysis and IHC, which may possess a better clinical value in developing novel therapies against recurrent EOC. The inhibition of Wnt signaling pathway was proved to suppress the CSC populations [49–51]. In recent years, several agents targeting the Wnt signaling pathway have been under clinical trials, including ipafricept, vantictumab, and CWP232291. Although these inhibitors may eliminate CSCs, it could affect the physiological processes in normal cells at the same time [52, 53]. The TGF-*β* signaling pathway maintains the homeostasis and quiescence of CSCs. Galunisertib (LY215729), one of TGF-*β* signaling pathway inhibitors, has been proved effective and safe for prostate cancer in a phase II study [52, 54]. An increasing number of drugs targeting CSCs are on the way to clinical application, and CSC targeting therapy joint with conventional chemotherapeutic treatment may be the next step closer to the cure of cancer in the future.

The enrichment of CSC signaling pathways in the chemoresistant relapse tumors was further validated in chemoresistant tumor cell line A2780cis (Figure 2(b), GSE33482). However, no OCSC enrichment was observed in another dataset using a closely related cell line Round5 A2780 (Figure 2(c), GSE15709). This may be attributed to the difference in cell line establishment methods between the two; only 5 rounds of cisplatin treatment were performed in the latter. In addition, no OCSC enrichment or statistical difference in CSC-related gene expression was observed between chemosensitive and chemoresistant primary tumor samples assessed by transcriptome analysis and IHC (Figures 2 and 3). These findings were inconsistent with the hypothesis that CSCs are seeds of chemoresistance. Aside from complex components of tumor tissues, rare CSCs exist among them. The relatively low sensitivity of transcriptome analysis may be the reason why no CSC enrichment was observed. Steg et al. reported that the expression level of OCSC markers significantly increased in post chemotherapy recurrent chemoresistant samples but not in recurrent samples without secondary chemotherapy [17]. It suggested that chemotherapy might be one of the factors that activate OCSCs. Based on these findings, it is possible that an increasing population of OCSCs can be found in recurrent tumor tissues from patients receiving multiline chemotherapy as the treatment going on. This growing number of residual OCSCs might gradually shorten the PFI in EOC patients. Moreover, different chemoresistant signal pathways were found in SP, a special type of CSCs, compared with CSCs isolated by surface markers. Only cellular response to drug was enriched in SP, while in surface marker identified CSCs, drug response, drug transport, cell cycle, and DNA damage response were all enriched (Figure 4). All these findings proved that chemoresistance is an inherent characteristic of OCSCs but also indicated that heterogeneity of CSCs should be taken into consideration when developing targeting strategies. Certainly, more thorough studies as well as more detailed information are needed to identify the relationship between OCSCs and chemoresistance in EOC relapse. Additionally, the inclusion of chemoresistant relapse samples from solid tumors other than ascites for comparative analysis might supply more consolidated evidence, which would minimize the bias caused by the differences in tumor microenvironment. In order to investigate the underlying mechanisms, extensive functional genomics experiments accompanied with sensitive transcriptome analysis are necessary, which will also reveal the potential specific CSC molecular targets to develop novel therapies against chemoresistant EOC recurrence.

## 5. Conclusions

In summary, the present study reported the potential activation of signaling pathways regulating pluripotency of stem cells in chemoresistant relapse tumors. The transcriptome analysis revealed upregulated CSC-related genes involved in acquired chemoresistance of EOC tumors. Our findings suggested the involvement of OCSCs in chemoresistant relapse of EOC and also indicated that these CSC DEGs correlated with acquired chemoresistance could become potential molecular targets to eliminate OCSCs in EOC. Based on the evidence, we further suggested the necessity to combine OCSC targeting therapy with conventional therapy in order to prevent chemoresistance as well as improve the prognosis of EOC patients.

## Figures and Tables

**Table 1:** KEGG pathways enriched in chemoresistant relapse and chemosensitive primary samples.

KEGG ID	Description	Count	*P*.adj value
*Upregulated pathways in chemoresistant relapse samples*			
hsa04657	IL-17 signaling pathway	25	1.03E-10
hsa04668	TNF signaling pathway	20	7.76E-06
hsa05205	Proteoglycans in cancer	26	1.84E-04
hsa05323	Rheumatoid arthritis	15	3.41E-04
hsa05134	Legionellosis	11	4.38E-04
hsa05144	Malaria	10	5.89E-04
hsa05120	Epithelial cell signaling in Helicobacter pylori infection	12	7.52E-04
hsa05202	Transcriptional misregulation in cancer	23	9.61E-04
hsa04725	Cholinergic synapse	16	9.70E-04
hsa04064	NF-kappa B signaling pathway	15	1.14E-03
hsa04010	MAPK signaling pathway	31	1.23E-03
hsa04060	Cytokine-cytokine receptor interaction	31	1.23E-03
hsa05020	Prion disease	29	1.56E-03
hsa04550	Signaling pathways regulating pluripotency of stem cells	18	1.92E-03
hsa05010	Alzheimer disease	36	2.06E-03
*Downregulated pathways in chemoresistant relapse samples*			
hsa04061	Viral protein interaction with cytokine and cytokine receptor	14	2.20E-03
hsa04933	AGE-RAGE signaling pathway in diabetic complications	14	2.20E-03
hsa05031	Amphetamine addiction	11	2.27E-03
hsa04390	Hippo signaling pathway	19	2.31E-03
hsa04216	Ferroptosis	8	2.44E-03
hsa05146	Amoebiasis	14	2.66E-03
hsa04115	p53 signaling pathway	11	3.58E-03
hsa04974	Protein digestion and absorption	26	3.76E-08
hsa04060	Cytokine-cytokine receptor interaction	42	8.67E-06
hsa04151	PI3K-Akt signaling pathway	44	1.19E-04
hsa04514	Cell adhesion molecules	25	1.19E-04
hsa04061	Viral protein interaction with cytokine and cytokine receptor	19	2.63E-04
hsa04512	ECM-receptor interaction	17	5.51E-04
hsa05340	Primary immunodeficiency	10	1.97E-03
hsa04510	Focal adhesion	26	4.24E-03
hsa04270	Vascular smooth muscle contraction	18	2.68E-02
hsa03010	Ribosome	20	2.68E-02
hsa04640	Hematopoietic cell lineage	14	4.84E-02

**Table 2:** GO biological process, cellular component (CC), and molecular function (MF) enriched in chemoresistant relapse and chemosensitive primary samples.

GO ID	Description	Ontology	Count	*P*.adj value
*Upregulated GO terms in chemoresistant relapse samples*				
GO:0008544	Epidermis development	BP	64	2.09E-07
GO:0032496	Response to lipopolysaccharide	BP	48	3.86E-06
GO:0043588	Skin development	BP	56	3.86E-06
GO:0002237	Response to molecule of bacterial origin	BP	48	9.33E-06
GO:0009913	Epidermal cell differentiation	BP	48	2.97E-05
GO:0071216	Cellular response to biotic stimulus	BP	35	1.59E-04
GO:0071496	Cellular response to external stimulus	BP	44	1.59E-04
GO:0007156	Homophilic cell adhesion via plasma membrane adhesion molecules	BP	28	1.59E-04
GO:0098742	Cell-cell adhesion via plasma-membrane adhesion molecules	BP	38	1.59E-04
GO:0048732	Gland development	BP	52	1.59E-04
GO:0048018	Receptor ligand activity	MF	59	5.43E-05
GO:0030546	Signaling receptor activator activity	MF	59	5.43E-05
GO:0001228	DNA-binding transcription activator activity, RNA polymerase II-specific	MF	49	3.37E-03
GO:0001216	DNA-binding transcription activator activity	MF	49	3.37E-03
GO:0005520	Insulin-like growth factor binding	MF	9	3.92E-03
GO:0046873	Metal ion transmembrane transporter activity	MF	48	4.40E-03
GO:0005125	Cytokine activity	MF	29	5.58E-03
GO:0033549	MAP kinase phosphatase activity	MF	6	1.54E-02
GO:0022836	Gated channel activity	MF	37	3.07E-02
GO:0005216	Ion channel activity	MF	44	3.26E-02
GO:0016755	Transferase activity, transferring amino-acyl groups	MF	6	3.56E-02
GO:0017017	MAP kinase tyrosine/serine/threonine phosphatase activity	MF	5	4.78E-02
*Downregulated GO terms in chemoresistant relapse samples*				
GO:0030198	Extracellular matrix organization	BP	73	3.42E-17
GO:0043062	Extracellular structure organization	BP	73	3.42E-17
GO:0061448	Connective tissue development	BP	48	9.05E-09
GO:0051216	Cartilage development	BP	39	1.07E-07
GO:0030199	Collagen fibril organization	BP	18	6.87E-07
GO:0002062	Chondrocyte differentiation	BP	27	1.44E-06
GO:0042110	T cell activation	BP	60	3.78E-06
GO:0003341	Cilium movement	BP	19	6.96E-06
GO:0001503	Ossification	BP	51	5.40E-05
GO:0061035	Regulation of cartilage development	BP	18	5.40E-05
GO:0062023	Collagen-containing extracellular matrix	CC	88	9.64E-25
GO:0005581	Collagen trimer	CC	33	1.66E-16
GO:0009897	External side of plasma membrane	CC	59	3.94E-09
GO:0005583	Fibrillar collagen trimer	CC	9	4.02E-08
GO:0098643	Banded collagen fibril	CC	9	4.02E-08
GO:0005604	Basement membrane	CC	22	1.80E-06
GO:0098644	Complex of collagen trimers	CC	10	1.91E-06
GO:0005788	Endoplasmic reticulum lumen	CC	44	2.61E-06
GO:0031514	Motile cilium	CC	28	1.04E-04
GO:0005930	Axoneme	CC	22	1.26E-04
GO:0005201	Extracellular matrix structural constituent	MF	49	1.91E-18
GO:0030020	Extracellular matrix structural constituent conferring tensile strength	MF	19	2.29E-10
GO:0019838	Growth factor binding	MF	30	2.25E-07
GO:0005518	Collagen binding	MF	19	2.44E-06
GO:0048407	Platelet-derived growth factor binding	MF	8	4.57E-06
GO:0019956	Chemokine binding	MF	12	2.69E-05
GO:0019957	C-C chemokine binding	MF	10	7.71E-05
GO:0016493	C-C chemokine receptor activity	MF	9	4.64E-04
GO:0005178	Integrin binding	MF	23	4.77E-04
GO:0048018	Receptor ligand activity	MF	55	5.66E-04

## Data Availability

The original contributions presented in the study are included in the article and supplementary material.

## Supplementary Materials

Supplementary Materials Supplementary Figure S1: the PCA score plots show a total of 39 samples in the ICGC AU-OV dataset. Three outlying samples were labelled. Supplementary Figure S2: the volcano plot of the differentially expressed genes in chemoresistant relapse samples. The threshold is ∣log2 fold change | >1 and adjusted *P* value < 0.05. The upregulated genes are shown in red, while the downregulated genes are shown in blue. Supplementary Figure S3: immunohistochemistry images of tumors from chemosensitive primary, chemoresistant primary, and chemoresistant relapse patients. The parts circled by the black boxes are shown in Figure 3. Magnification 200x and scale bar = 200 *μ*m. Supplementary Table S1: the clinical information of the 39 samples from ICGC OV-AU dataset. Supplementary Table S2: the detailed information of 8 GEO datasets. Supplementary Table S3: the clinical information of 11 ovarian cancer patients. Supplementary Table S4: the detailed information of 4 antibodies used in IHC. Supplementary Table S5: the 25 CSC-related genes.
