# Supplementary material for: Involvement of Cancer Stem Cells in Chemoresistant Relapse of Epithelial Ovarian Cancer Identified by Transcriptome Analysis
Source: J Oncol. 2022 Mar 31;2022:6406122. doi: 10.1155/2022/6406122 (PMC8991408; doi:10.1155/2022/6406122)
Supplement: Supplementary Materials — Supplementary Figure S1: the PCA score plots show a total of 39 samples in the ICGC AU-OV dataset. Three outlying samples were labelled. Supplementary Figure S2: the volcano plot of the differentially expressed genes in chemoresistant relapse samples. The threshold is ∣log2 fold change | >1 and adjusted P value < 0.05. The upregulated genes are shown in red, while the downregulated genes are shown in blue. Supplementary Figure S3: immunohistochemistry images of tumors from chemosensitive primary, chemoresistant primary, and chemoresistant relapse patients. The parts circled by the black boxes are shown in Figure 3. Magnification 200x and scale bar = 200 μm. Supplementary Table S1: the clinical information of the 39 samples from ICGC OV-AU dataset. Supplementary Table S2: the detailed information of 8 GEO datasets. Supplementary Table S3: the clinical information of 11 ovarian cancer patients. Supplementary Table S4: the detailed information of 4 antibodies used in IHC. Supplementary Table S5: the 25 CSC-related genes. [file 6406122.f1.zip › 6406122.f2.docx]

**Supplementary Table S1: The clinical information of the 39 samples from ICGC OV-AU dataset.**

| **Donor ID** | **Age** | **Tumor Stage**  **（FIGO）** | **Sample ID** | **Sample Type** | **Chemotherapy response** |
| --- | --- | --- | --- | --- | --- |
| AOCS-034 | 52 | III | AOCS-034-2-4 | Primary tumor - solid tissue | Sensitive |
|  |  |  | AOCS-034-4-1 | Recurrent tumor - ascitic fluid | Resistant |
| AOCS-064 | 67 | III | AOCS-064-2-X | Primary tumor - solid tissue | Sensitive |
|  |  |  | AOCS-064-4-7 | Recurrent tumor - ascitic fluid | Resistant |
| AOCS-065 | 46 | III | AOCS-065-2-2 | Primary tumor - solid tissue | Sensitive |
|  |  |  | AOCS-065-4-X | Recurrent tumor - ascitic fluid | Resistant |
| AOCS-086 | 65 | III | AOCS-086-2-9 | Primary tumor - solid tissue | Sensitive |
|  |  |  | AOCS-086-4-6 | Recurrent tumor - ascitic fluid | Resistant |
| AOCS-088 | 57 | III | AOCS-088-2-4 | Primary tumor - solid tissue | Sensitive |
|  |  |  | AOCS-088-4-1 | Recurrent tumor - ascitic fluid | Resistant |
| AOCS-090 | 54 | III | AOCS-090-2-4 | Primary tumor - solid tissue | Sensitive |
|  |  |  | AOCS-090-4-1 | Recurrent tumor - ascitic fluid | Resistant |
| AOCS-091 | 39 | III | AOCS-091-2-7 | Primary tumor - solid tissue | Sensitive |
|  |  |  | AOCS-091-4-4 | Recurrent tumor -ascitic fluid | Resistant |
| AOCS-092 | 69 | III | AOCS-092-2-X | Primary tumor - solid tissue | Sensitive |
|  |  |  | AOCS-092-4-7 | Recurrent tumor - ascitic fluid | Resistant |
| AOCS-093 | 58 | III | AOCS-093-10-1 | Primary tumor - ascitic fluid | Sensitive |
|  |  |  | AOCS-093-2-2 | Primary tumor - solid tissue | Sensitive |
|  |  |  | AOCS-093-4-X | Recurrent tumor - ascitic fluid | Resistant |
| AOCS-094 | 59 | III | AOCS-094-2-5 | Primary tumor - solid tissue | Sensitive |
|  |  |  | AOCS-094-4-2 | Recurrent tumor - solid tissue | Resistant |
| AOCS-095 | 51 | III | AOCS-095-2-8 | Primary tumor - solid tissue | Sensitive |
|  |  |  | AOCS-095-4-5 | Recurrent tumor - ascitic fluid | Resistant |
| AOCS-117 | 54 | III | AOCS-117-4-7 | Recurrent tumor - ascitic fluid | Resistant |
| AOCS-119 | 65 | III | AOCS-119-4-2 | Recurrent tumor - ascitic fluid | Resistant |
| AOCS-120 | 47 | III | AOCS-120-4-X | Recurrent tumor - ascitic fluid | Resistant |
| AOCS-134 | 46 | III | AOCS-134-4-2 | Recurrent tumor - ascitic fluid | Resistant |
| AOCS-135 | 52 | III | AOCS-135-10-7 | Recurrent tumor - ascitic fluid | Resistant |
|  |  |  | AOCS-135-4-5 | Recurrent tumor - ascitic fluid | Resistant |
| AOCS-137 | 68 | IV | AOCS-137-10-2 | Recurrent tumor - ascitic fluid | Resistant |
|  |  |  | AOCS-137-4-0 | Primary tumor - ascitic fluid | Sensitive |
| AOCS-138 | 57 | III | AOCS-138-4-3 | Recurrent tumor - ascitic fluid | Resistant |
| AOCS-139 | 62 | IV | AOCS-139-2-9 | Primary tumor - solid tissue | Sensitive |
| AOCS-141 | 62 | III | AOCS-141-10-8 | Recurrent tumor - ascitic fluid | Resistant |
|  |  |  | AOCS-141-4-6 | Recurrent tumor - ascitic fluid | Resistant |
| AOCS-142 | 71 | III | AOCS-142-4-9 | Recurrent tumor - ascitic fluid | Resistant |
| AOCS-150 | 55 | III | AOCS-150-10-7 | Recurrent tumor - ascitic fluid | Resistant |
| AOCS-155 | 69 | III | AOCS-155-4-9 | Recurrent tumor - ascitic fluid | Resistant |
| AOCS-167 | 49 | IV | AOCS-167-4-6 | Recurrent tumor - ascitic fluid | Resistant |
