# Supplementary material for: Involvement of Cancer Stem Cells in Chemoresistant Relapse of Epithelial Ovarian Cancer Identified by Transcriptome Analysis
Source: J Oncol. 2022 Mar 31;2022:6406122. doi: 10.1155/2022/6406122 (PMC8991408; doi:10.1155/2022/6406122)
Supplement: Supplementary Materials — Supplementary Figure S1: the PCA score plots show a total of 39 samples in the ICGC AU-OV dataset. Three outlying samples were labelled. Supplementary Figure S2: the volcano plot of the differentially expressed genes in chemoresistant relapse samples. The threshold is ∣log2 fold change | >1 and adjusted P value < 0.05. The upregulated genes are shown in red, while the downregulated genes are shown in blue. Supplementary Figure S3: immunohistochemistry images of tumors from chemosensitive primary, chemoresistant primary, and chemoresistant relapse patients. The parts circled by the black boxes are shown in Figure 3. Magnification 200x and scale bar = 200 μm. Supplementary Table S1: the clinical information of the 39 samples from ICGC OV-AU dataset. Supplementary Table S2: the detailed information of 8 GEO datasets. Supplementary Table S3: the clinical information of 11 ovarian cancer patients. Supplementary Table S4: the detailed information of 4 antibodies used in IHC. Supplementary Table S5: the 25 CSC-related genes. [file 6406122.f1.zip › 6406122.f3.docx]

**Supplementary Table S2: The detailed information of 8 GEO datasets.**

| **GEO** | **Samples** | **GPL** | **Citation** |
| --- | --- | --- | --- |
| GSE33482 | A2780 (n=6）  A2780cis (n=6) | GPL6480 | Feilotter et al. [1] |
| GSE15709 | Parental A2780 (n=5)  Round5 A2780 (n=5) | GPL570 | Li et al. [2] |
| GSE28739 | Primary chemosensitive (n=20)  Primary chemoresistant(n=30) | GPL7264 | Trinh et al. [3] |
| GSE51373 | Chemoresistant (n=12)  Chemosensitive (n=16) | GPL570 | Koti et al. [4] |
| GSE131978 | Platinum sensitive (n=5)  Platinum resistant (n=7) | GPL570 | Tassi et al. [5] |
| GSE28799 | Monolayer OVCAR3 (n=3)  Spheroid OVCAR3 (n=3) | GPL570 | Wang et al. [6] |
| GSE82304 | ALDH-low SKOV3 (n=3)  ALDH-high SKOV3 (n=3) | GPL10558 | Sharrow et al. [7] |
| GSE33874 | Hoechst33342 dye sorted main population MP (n=10)  Hoechst33342 dye sorted side population SP (n=10) | GPL570 | Vathipadiekal et al. [8] |

1. Haslehurst, A.M., et al., *EMT transcription factors snail and slug directly contribute to cisplatin resistance in ovarian cancer.* BMC Cancer, 2012. **12**: p. 91.

2. Li, M., et al., *Integrated analysis of DNA methylation and gene expression reveals specific signaling pathways associated with platinum resistance in ovarian cancer.* BMC Med Genomics, 2009. **2**: p. 34.

3. Trinh, X.B., et al., *Microarray-based oncogenic pathway profiling in advanced serous papillary ovarian carcinoma.* PLoS One, 2011. **6**(7): p. e22469.

4. Koti, M., et al., *Identification of the IGF1/PI3K/NF kappaB/ERK gene signalling networks associated with chemotherapy resistance and treatment response in high-grade serous epithelial ovarian cancer.* BMC Cancer, 2013. **13**: p. 549.

5. Tassi, R.A., et al., *FXYD5 (Dysadherin) upregulation predicts shorter survival and reveals platinum resistance in high-grade serous ovarian cancer patients.* Br J Cancer, 2019. **121**(7): p. 584-592.

6. Wang, L., et al., *Isolation and characterization of stem-like cells from a human ovarian cancer cell line.* Mol Cell Biochem, 2012. **363**(1-2): p. 257-68.

7. Sharrow, A.C., et al., *Characterization of aldehyde dehydrogenase 1 high ovarian cancer cells: Towards targeted stem cell therapy.* Gynecol Oncol, 2016. **142**(2): p. 341-8.

8. Vathipadiekal, V., et al., *Identification of a potential ovarian cancer stem cell gene expression profile from advanced stage papillary serous ovarian cancer.* PLoS One, 2012. **7**(1): p. e29079.
