# Supplementary material for: Involvement of Cancer Stem Cells in Chemoresistant Relapse of Epithelial Ovarian Cancer Identified by Transcriptome Analysis
Source: J Oncol. 2022 Mar 31;2022:6406122. doi: 10.1155/2022/6406122 (PMC8991408; doi:10.1155/2022/6406122)
Supplement: Supplementary Materials — Supplementary Figure S1: the PCA score plots show a total of 39 samples in the ICGC AU-OV dataset. Three outlying samples were labelled. Supplementary Figure S2: the volcano plot of the differentially expressed genes in chemoresistant relapse samples. The threshold is ∣log2 fold change | >1 and adjusted P value < 0.05. The upregulated genes are shown in red, while the downregulated genes are shown in blue. Supplementary Figure S3: immunohistochemistry images of tumors from chemosensitive primary, chemoresistant primary, and chemoresistant relapse patients. The parts circled by the black boxes are shown in Figure 3. Magnification 200x and scale bar = 200 μm. Supplementary Table S1: the clinical information of the 39 samples from ICGC OV-AU dataset. Supplementary Table S2: the detailed information of 8 GEO datasets. Supplementary Table S3: the clinical information of 11 ovarian cancer patients. Supplementary Table S4: the detailed information of 4 antibodies used in IHC. Supplementary Table S5: the 25 CSC-related genes. [file 6406122.f1.zip › 6406122.f4.docx]

**Supplementary Table S3: The clinical information of 11 ovarian cancer patients.**

| **Patients** | **Tissue Type** | **Pathology** | **Stage** | **Age** | **Treatment** | **Chemotherapy Response** |
| --- | --- | --- | --- | --- | --- | --- |
| 18-43582 | Primary tumor | Serous | Ⅱa | 66 | cisplatin and paclitaxel 1 cycle,  carboplatin and paclitaxel 5 cycles | sensitive |
| 17-32137 | Primary tumor | Serous | Ⅰc | 48 | cisplatin and paclitaxel 5 cycles | sensitive |
| 18-38772 | Primary tumor | Serous | Ⅰc | 63 | cisplatin and paclitaxel 6 cycles | sensitive |
| 17-36954 | Primary tumor | Serous | Ⅲc | 53 | cisplatin and paclitaxel 6 cycles | sensitive |
| 16-17712 | Primary tumor | Serous | Ⅲc | 40 | cisplatin and paclitaxel 8 cycles | sensitive |
| 18-47358 | Primary tumor | Serous | Ⅲc | 49 | cisplatin and paclitaxel 6 cycles,  carboplatin and paclitaxel 4 cycles | resistant |
| 19-21613 | Primary tumor | Serous | Ⅲc | 53 | cisplatin and paclitaxel 3 cycles,  carboplatin and paclitaxel 3 cycles | resistant |
| 17-13474 | Primary tumor | Serous | Ⅲc | 53 | carboplatin and paclitaxel 8 cycles | resistant |
| 18-11049 | Primary tumor | Serous | Ⅲc | 61 | cisplatin and paclitaxel 7 cycles, | resistant |
| 17-17656/  19-33326 | Primary tumor & relapse tumor | Serous | Ⅲb | 57 | primary: carboplatin and paclitaxel 6 cycles | sensitive |
|  |  |  |  |  | relapse: cisplatin and paclitaxel 6 cycles | resistant |
| 19-54632 | Relapse tumor | Serous | unknown | 48 | primary: platinum-based chemotherapy | sensitive |
|  |  |  |  |  | relapse: cisplatin and gemcitabine 3 cycles, gemcitabine and bevacizumab 2 cycles, gemcitabine and bevacizumab and cisplatin 1 cycle, carboplatin and paclitaxel and bevacizumab 2 cycles | resistant |
