# Supplementary material for: Involvement of Cancer Stem Cells in Chemoresistant Relapse of Epithelial Ovarian Cancer Identified by Transcriptome Analysis
Source: J Oncol. 2022 Mar 31;2022:6406122. doi: 10.1155/2022/6406122 (PMC8991408; doi:10.1155/2022/6406122)
Supplement: Supplementary Materials — Supplementary Figure S1: the PCA score plots show a total of 39 samples in the ICGC AU-OV dataset. Three outlying samples were labelled. Supplementary Figure S2: the volcano plot of the differentially expressed genes in chemoresistant relapse samples. The threshold is ∣log2 fold change | >1 and adjusted P value < 0.05. The upregulated genes are shown in red, while the downregulated genes are shown in blue. Supplementary Figure S3: immunohistochemistry images of tumors from chemosensitive primary, chemoresistant primary, and chemoresistant relapse patients. The parts circled by the black boxes are shown in Figure 3. Magnification 200x and scale bar = 200 μm. Supplementary Table S1: the clinical information of the 39 samples from ICGC OV-AU dataset. Supplementary Table S2: the detailed information of 8 GEO datasets. Supplementary Table S3: the clinical information of 11 ovarian cancer patients. Supplementary Table S4: the detailed information of 4 antibodies used in IHC. Supplementary Table S5: the 25 CSC-related genes. [file 6406122.f1.zip › 6406122.f5.docx]

**Supplementary Table S4: The detailed information of 4 antibodies used in IHC.**

| **Name** | **Brand** | **Catalog Number** | **Host Species** | **Reactivity** | **Concentration** |
| --- | --- | --- | --- | --- | --- |
| Anti-KLF4 antibody | Abcam | #ab215036 | Rabbit | Human | 1:2000 |
| Anti-PAX6 antibody | Abcam | #ab195045 | Rabbit | Mouse, Rat, Human | 1:500 |
| Anti-SMAD3 antibody | Abcam | #ab40854 | Rabbit | Rat, Human | 1:500 |
| Anti-Wnt3a antibody | Abcam | #ab219412 | Rabbit | Mouse, Rat, Human | 1:500 |
