# Supplementary material for: Involvement of Cancer Stem Cells in Chemoresistant Relapse of Epithelial Ovarian Cancer Identified by Transcriptome Analysis
Source: J Oncol. 2022 Mar 31;2022:6406122. doi: 10.1155/2022/6406122 (PMC8991408; doi:10.1155/2022/6406122)
Supplement: Supplementary Materials — Supplementary Figure S1: the PCA score plots show a total of 39 samples in the ICGC AU-OV dataset. Three outlying samples were labelled. Supplementary Figure S2: the volcano plot of the differentially expressed genes in chemoresistant relapse samples. The threshold is ∣log2 fold change | >1 and adjusted P value < 0.05. The upregulated genes are shown in red, while the downregulated genes are shown in blue. Supplementary Figure S3: immunohistochemistry images of tumors from chemosensitive primary, chemoresistant primary, and chemoresistant relapse patients. The parts circled by the black boxes are shown in Figure 3. Magnification 200x and scale bar = 200 μm. Supplementary Table S1: the clinical information of the 39 samples from ICGC OV-AU dataset. Supplementary Table S2: the detailed information of 8 GEO datasets. Supplementary Table S3: the clinical information of 11 ovarian cancer patients. Supplementary Table S4: the detailed information of 4 antibodies used in IHC. Supplementary Table S5: the 25 CSC-related genes. [file 6406122.f1.zip › 6406122.f6.docx]

**Supplementary Table S5. The 25 CSCs-related genes.**

| **Gene symbol** | **Descriptions** |
| --- | --- |
| **Signaling pathways regulating pluripotency of stem cells** | |
| KLF4 | Kruppel Like Factor 4 |
| SMAD3 | SMAD Family Member 3 |
| WNT3 | Wnt Family Member 3 |
| PAX6 | Paired Box 6 |
| NODAL | Nodal Growth Differentiation Factor |
| ID1 | Inhibitor of DNA Binding 1, HLH Protein |
| LIF | Leukemia Inhibitory Factor |
| APC2 | APC Regulator of WNT Signaling Pathway 2 |
| KRAS | KRAS Proto-Oncogene, GTPase |
| WNT7B | Wnt Family Member 7B |
| ID2 | Inhibitor of DNA Binding 2 |
| HOXA1 | Homeobox A1 |
| MAPK12 | Mitogen-Activated Protein Kinase 12 |
| FZD9 | Frizzled Class Receptor 9 |
| WNT3A | Wnt Family Member 3A |
| FGFR3 | Fibroblast Growth Factor Receptor 3 |
| OTX1 | Orthodenticle Homeobox 1 |
| WNT16 | Wnt Family Member 16 |
| **Cancer stem cell surface markers** | |
| CD44 | CD44 Molecule (Indian Blood Group) |
| KIT | KIT Proto-Oncogene, Receptor Tyrosine Kinase (CD117) |
| PROM1 | Prominin 1 (CD133) |
| ALDH1A1 | Aldehyde Dehydrogenase 1 Family Member A1 |
| **Stem cell transcription factors** | |
| SOX2 | SRY-Box Transcription Factor 2 |
| POU5F1 | POU Class 5 Homeobox 1 (OCT4) |
| NANOG | Nanog Homeobox |
